# Pulmonary Embolism Presenting as Abdominal Pain: An Atypical Presentation of a Common Diagnosis

**DOI:** 10.1155/2016/7832895

**Published:** 2016-08-24

**Authors:** Hasan Rehman, Elizabeth John, Payal Parikh

**Affiliations:** ^1^Department of Medicine, Rutgers Robert Wood Johnson Medical School, New Brunswick, NJ 08901, USA; ^2^Department of Medicine, University Medical Center of Princeton at Plainsboro, Plainsboro, NJ 08536, USA

## Abstract

Pulmonary embolism (PE) is a frequent diagnosis made in the emergency department and can present in many different ways. Abdominal pain is an unusual presenting symptom for PE. It is essential to maintain a high degree of suspicion in these patients, as a delay in diagnosis can be devastating for the patient and confers a high risk of mortality if left untreated. Here, we report the case of a 53-year-old male who presented to the emergency department with worsening right upper quadrant abdominal pain with fevers. Initial imaging was benign, although lab work showed worsening leukocytosis and bilirubin. Abdominal pathology seemed most likely, but the team kept PE on the differential. Further imaging revealed acute pulmonary embolus in the segmental branch of the right lower lobe extending distally into subsegmental branches. The patient was started on anticoagulation and improved drastically. This case highlights the necessity of keeping a broad differential and maintaining a systematic approach when dealing with nonspecific complaints. Furthermore, a discussion on the pathophysiology on why PE can present atypically as abdominal pain, as well as fevers, is reviewed. Using this information can hopefully lead to a subtle diagnosis of PE in the future and lead to a life-saving diagnosis.

## 1. Introduction

Pulmonary embolism (PE) is a commonly encountered diagnosis in the emergency room. Given the devastating effects of a high clot burden, it is imperative that a diagnosis is made and treatment is initiated promptly. There is a very high risk of morbidity and mortality if there is a delay in treatment. However, a PE can present in many different ways so a high clinical suspicion is necessary to avoid misdiagnosis. Abdominal pain, in particular, is a very uncommon presenting symptom for PE [1]. Herein, we report the case of a 53-year-old male who presented with worsening right upper quadrant abdominal pain, later found to have a PE.

## 2. Case Report

Patient is a 53-year-old male with history of hypertension who presents with two days of sudden onset of abdominal pain. He reports that pain began on his lower right back and radiated over to the right upper quadrant of his abdomen. Pain is described as constant dullness in the area with periodic bouts of sharp pain. Eating and deep inspiratory breaths exacerbate symptoms. He also notes intermittent fevers during this time with temperatures ranging within 100.8–102 F. Patient denies any chest pain, dyspnea, chills, sick contacts, or previous history of these symptoms. The patient did not recently travel and had no recent surgeries or history of clots. There was no other significant medical history except for acute pancreatitis at age 15 without known cause. Patient does not take any medications. Family history is unremarkable with no known clotting disorders or malignancy. Social history is benign, with no reported cigarette, alcohol, or drug use.

On admission, patient had a temperature of 100.2 F, blood pressure of 137/83, with pulse of 100, respiratory rate of 22, and O_2_ saturation of 96% on room air. Physical exam revealed a well-nourished male who appeared mildly uncomfortable on bed, with no evidence of JVD or calf tenderness/erythema. Heart was tachycardic, but otherwise normal with no rubs, murmurs, or gallops. Lungs were clear to auscultation bilaterally with no rales, rhonchi, or wheezes noted. Abdomen was tender in the right upper quadrant, but soft and nondistended. No evidence of rebound or guarding was appreciated on exam. Murphy's sign was negative.

**Figure 1:**
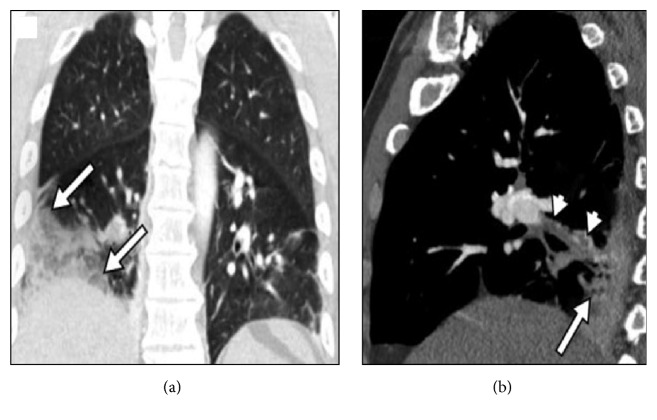
(a) Coronal CTA image in lung window shows infiltrate in the right lung base, suggestive of a peripherally located infarcted lung (arrows). (b) Sagittal CTA image shows embolism within the right lower lobe segmental branches (arrowheads) and the peripherally located infarcted lung (arrow) [10].

**Figure 2:**
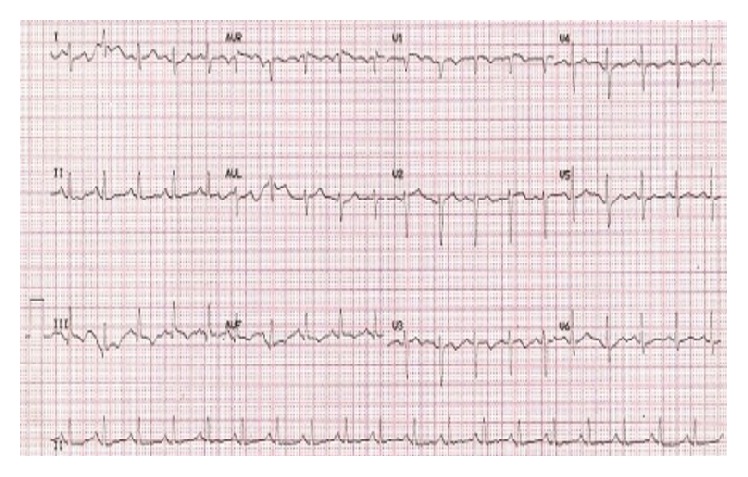
EKG with classic findings of S1Q3T3, an s wave in lead 1, q wave in lead 3, and T wave inversion in lead 3. Reference: EKG Electrophysiology Library of Dr. Johnson Francis.

Significant labs included a white count of 12.1, liver function tests within normal limits, bilirubin of 1.6, and troponins negative times two. Right upper quadrant ultrasound showed no evidence of gallstones, no gallbladder distention, negative sonographic Murphy's sign, and no biliary dilation. CT scan of the abdomen/pelvis was read as small right pleural effusion with atelectasis at the right base, with no evidence of acute intra-abdominal process. CTA of the chest revealed an acute pulmonary embolus in the segmental branch of the right lower lobe extending distally into subsegmental branches; the infiltrate in the right lung base most likely represents infarcted lung (Figure 1). EKG performed the following day demonstrated sinus rhythm, with classic findings consistent with S1Q3T3 (Figure 2) [2]. Patient was subsequently started on therapeutic enoxaparin. However, abdominal pain continued to worsen and bilirubin rose to 2.2 the next day. Pain seemed to be out of proportion to referred pain from the pulmonary embolus. Given continued low-grade fevers, zosyn was initiated. Visceral ultrasound of the abdomen was performed to rule out portal vein thrombosis, which was negative. Direct and indirect bilirubin was measured which showed direct bilirubin of 0.5, consistent with indirect hyperbilirubinemia. Subsequent hemolysis labs were negative. Patient eventually transitioned to rivaroxaban and abdominal pain slowly subsided. He was discharged on rivaroxaban with close outpatient follow-up.

## 3. Discussion

This case illustrates the necessity of having a high index of suspicion for pulmonary embolism even in patients presenting with nonclassical symptoms. This patient had a convincing story for abdominal etiology, as evidenced by elevated bilirubin, increased white blood cell count, and low-grade fever. However, the pleuritic pain elicited on history as well as tachycardia and tachypnea kept pulmonary embolism on the differential. Given these findings, further workup was pursued, leading to the subtle diagnosis.

Many hypotheses have been proposed regarding the mechanism of action explaining abdominal pain in the setting of a pulmonary embolism. It has been postulated that right heart strain from the clot could cause backflow, leading to passive hepatic congestion. Furthermore, distension of Glisson's capsule resulting from the congestion could lead to the presenting symptoms [3]. However, in this patient, there was no clinical or physical exam evidence of right heart strain. In addition, echocardiogram was within normal limits. Additionally, the abdominal pain may be related to diaphragmatic pleurisy [4]. This is thought to result from pulmonary infarction in the lung bases distally to the area of the clot [5]. Lastly, it has been suggested that the pain is related to tension on sensory nerve endings in the parietal pleura as an effect of the burden on the clot. This area innervates the intercostal muscles, leading to pain in this area [6].

The presentation was further clouded by fevers and worsening bilirubin. Low-grade fevers have long been studied as a well-known phenomenon accompanying PE [7]. This has been suggested to be secondary to infarction resulting in tissue necrosis and local inflammation [8]. It is not unusual to have concomitant leukocytosis, as seen here [9]. The degree of fever seen in our patient coexisting with worsening abdominal pain was a bit curious, however, and prompted the decision on starting empiric antibiotics. The increased bilirubin also questioned the diagnosis of PE and made an abdominal pathology more likely. Although the bilirubin initially rose, it eventually trended downwards; thus, it was suspected that the patient might have had Gilbert's disease, with the increase likely secondary to the stress of the PE.

## 4. Conclusion

Here, we describe an interesting case of PE that could have easily been missed without a high index of suspicion. Missing such a diagnosis can lead to devastating consequences. A variety of nonspecific complaints, such as abdominal pain, can mask the underlying disease. Many other labs seen here also confounded the picture. However, maintaining a systematic approach and recognizing the different signs, symptoms, and laboratory findings in PE can lead to a life-saving diagnosis.
